# Crohn disease and schizophrenia: fortuitous association or etiopathogenic link?

**DOI:** 10.1192/j.eurpsy.2023.1587

**Published:** 2023-07-19

**Authors:** B. Zineb, T. Aicha, K. Imane, L. Fouad, O. Abderrazak

**Affiliations:** 1 Ar-razi Psychiatric hospital, Faculty of Medecine and Pharmacy, Rabat, Morocco

## Abstract

**Introduction:**

Crohn’s disease is a chronic inflammatory bowel disease of multifactorial etiology. Its association with psychiatric disorders has frequently been reported, mainly with depressive or anxiety disorders.However, its association with schizophrenia remains exceptional.

**Objectives:**

we will try to discuss this association.

**Methods:**

In this regard, we report the case of a young patient, aged 24, diagnosed 5 years ago with Crohn’s disease, evolving by remission flares, currently treated with Azathioprine, after failure of corticosteroid bolus.

**Results:**

The patient was admitted to psychiatry for aggression towards his parents.In view of the history of the disorders as reported by the family and the psychiatric interview, the diagnosis of schizophrenia was retained and the patient was put on amisulpride.

**Conclusions:**

Schizophrenia and Crohn’s disease are relatively frequent diseases, generally occurring at a young age, whose etiopathogenesis, multifactorial, involves in both cases genetic, environmental and immunological factors.Their association does not seem fortuitous and arouses both etiopathogenic and therapeutic interest, but studies involving a large number of patients would make it possible to elucidate the link between these two diseases.

**Disclosure of Interest:**

None Declared

